# Prime–Boost with *Mycobacterium smegmatis* Recombinant Vaccine Improves Protection in Mice Infected with *Mycobacterium tuberculosis*

**DOI:** 10.1371/journal.pone.0078639

**Published:** 2013-11-08

**Authors:** Ana Paula Junqueira-Kipnis, Fábio Muniz de Oliveira, Monalisa Martins Trentini, Sangeeta Tiwari, Bing Chen, Danilo Pires Resende, Bruna D. S. Silva, Mei Chen, Lydia Tesfa, William R. Jacobs, André Kipnis

**Affiliations:** 1 Instituto de Patologia Tropical e Saúde Pública. Universidade Federal de Goiás, Goiânia, Goiás, Brazil; 2 Microbiology and Immunology, Molecular Genetics, Albert Einstein College of Medicine, New York, New York, United States of America; 3 Flow Cytometry Core Facility, Albert Einstein College of Medicine, New York, New York, United States of America; University of Delhi, India

## Abstract

The development of a new vaccine as a substitute for Bacillus Calmette–Guerin or to improve its efficacy is one of the many World Health Organization goals to control tuberculosis. Mycobacterial vectors have been used successfully in the development of vaccines against tuberculosis. To enhance the potential utility of *Mycobacterium smegmatis* as a vaccine, it was transformed with a recombinant plasmid containing the partial sequences of the genes *Ag85c*, *MPT51*, and *HspX* (CMX) from *M. tuberculosis*. The newly generated recombinant strain mc^2^-CMX was tested in a murine model of infection. The recombinant vaccine induced specific IgG1 or IgG2a responses to CMX. CD4^+^ and CD8^+^ T cells from the lungs and spleen responded *ex vivo* to CMX, producing IFN-γ, IL17, TNF-α, and IL2. The vaccine thus induced a significant immune response in mice. Mice vaccinated with mc^2^-CMX and challenged with *M. tuberculosis* showed better protection than mice immunized with wild-type *M. smegmatis* or BCG. To increase the safety and immunogenicity of the CMX antigens, we used a recombinant strain of *M. smegmatis*, IKE (immune killing evasion), to express CMX. The recombinant vaccine IKE-CMX induced a better protective response than mc^2^-CMX. The data presented here suggest that the expression of CMX antigens improves the immune response and the protection induced in mice when *M. smegmatis* is used as vaccine against tuberculosis.

## Introduction

Tuberculosis (TB) was the eighth most frequent cause of death worldwide in 2008 according to a World Health Organization (WHO) report, although since the Millennium Development Goals and Stop TB Strategy were established by WHO, more than 51 million people have been successfully treated and cured of TB [1]. Although these data are encouraging, the incidence of the disease is persistently high in several countries, explaining the continual spread of TB.

Early diagnostic and prophylactic strategies are still required to control TB. Bacillus Calmette–Guerin (BCG), the currently available vaccine for TB, although safe, does not protect against TB in adults [2]. To date, several TB vaccines have been tested in preclinical and clinical trials and in 2012, a review article described 12 promising vaccines. Those vaccines differ in their vaccination schemes and their use as primary vaccines or boosters for the BCG vaccine. The only vaccine being tested in a phase 3 trial to date is an attenuated *Mycobacterium indicus pranii*, to be used in immunotherapy [3].

To select molecules or agents that can be tested as TB vaccines, it is important to understand the immune response induced during TB infection. TB has several clinical forms, and approximately one third of the world population is infected with *M. tuberculosis* (*Mtb*), comprising a massive population of individuals with latent disease. It has been shown by our group and others that the immune response of individuals infected with *Mtb* specifically recognizes a set of proteins, including HspX, produced by *Mtb* bacilli under stress conditions that mimic *in vivo* circumstances [4]–[7]. Patients that present with active pulmonary TB, for instance, preferentially recognize and respond to other *Mtb* antigens, i.e., the GLc-B, MPT51, Ag85 complex [8]–[12]. Some responses in patients with extrapulmonary forms of TB are the same as those seen with the active pulmonary TB form [12], [13]. These features support the hypothesis that a good candidate vaccine should induce immune responses to antigens common to more than one form of TB and should include more than one protein in a single formulation. Antigens recognized by both the humoral and cellular immune responses might also improve the protection profile of a vaccine [14], [15].

Several proteins from *Mtb have been* tested as subunit vaccines or in recombinant vectors, and those vaccines composed of more than one protein/antigen showed sufficient potential to proceed to clinical assays [3]. Recently, we showed that a fusion protein construct (CMX) containing the immune epitopes of antigens 85 c and MPT51 and the entire HspX antigen from *Mtb* was immunogenic in mice and antigenic in individuals with active TB, confirming its potential utility as a vaccine for TB [15]. Because *M. smegmatis* is attenuated, grows rapidly, can express *Mtb* antigens, and has been shown to induce enhanced protection against TB in a murine model of infection, we used *M. smegmatis* expressing CMX as a recombinant vaccine.

## Materials and Methods

### Ethics Statement

This study was carried out in strict accordance with the recommendations in the Guide for the Care and Use of Laboratory Animals of the Committee of Sociedade Brasileira de Animais de Laboratório (SBAL). The protocol for this work was approved by the Committee on the Ethics of Animal Experiments of the University Federal de Goiás (permit number: 229/11).

### Bacterial Strains, Plasmids, and Vaccine Construction

The *M. smegmatis* (mc^2^) strains used in this study were derived from laboratory strain mc^2^155. During the study, the strains were grown in 7H9 liquid medium containing 10% Oleic acid, dextrose and catalase (OADC), 0.5% glycerol, and 0.05% Tween 80 for maintenance, transformation, and transduction.

Recombinant CMX–*M. smegmatis* (mc^2^-CMX) construction. A DNA sequence encoding the *Ag85c* and *MPT51* immunodominant epitopes and the entire *HspX* gene, described in a previous study [15], was used as the template for PCR amplification using a set of primers that allowed the creation of flanking restriction enzyme sites and facilitated posterior cloning. The product of this amplification was cloned into the pGEM-T Easy vector (Promega, USA). The recombinant pGEM-T Easy vector containing the fused gene sequence (CMX) was digested with KpnI and *Not*I to remove the CMX fragment. The digested fragment was then ligated into the *Mycobacterium*/*Escherichia coli* shuttle vector pLA71 [16], previously digested with the same enzymes, and resolved on agarose gel to remove the *PhoA* gene. The recombinant pLA71/CMX vector was sequenced to check for mutations. The pLA71/CMX and pLA71 vectors were transformed into mc^2^ cells and screened on medium containing kanamycin, to generate the recombinant mc^2^-CMX strain and the mc^2^ control vaccine, respectively.

Immune killing evasion (IKE) *M. smegmatis* mutant construction. The *M. smegmatis* mutant, mc^2^6462 or IKE (mc^2^155Δ 0615–0626) was constructed by homologous recombination using a specialized transducing phage [17], [18]. The deletion phagemid for the mutant strain mc^2^6462 was constructed by PCR amplification of the 5′-flanking and 3′-flanking regions of the genes to be knocked out, and then cloned into p0004s and packaged into the temperature-sensitive phage ph159. This phage was used to generate the knockout strain using specialized transduction, as described previously [19]. The clones obtained were confirmed by PCR and Southern analysis. Then mc^2^6463, an unmarked strain, was generated from mc^2^6462 by transducing the latter with phage phAE280 containing resolvase, to remove the selection and counterselection markers, hygromycin and SacB, after plating on 7H10 plates containing 5% sucrose. The medium was supplemented with 50 µg/mL hygromycin, 20 µg/mL apramycin, or 20 µg/mL kanamycin when required.

### SDS–PAGE and Western Blotting

Western blotting was performed to detect recombinant CMX protein expression. The SDS-PAGE-separated proteins were electrotransferred to a nitrocellulose membrane. A 1∶10,000 dilution of polyclonal antiserum, obtained from BALB/c mice after three vaccinations with rCMX and complete Freund’s adjuvant were preadsorbed with *E. coli* proteins, and then incubated with the nitrocellulose membrane. The membrane was then incubated with horseradish-peroxidase-conjugated anti-mouse IgG (Sigma-Aldrich®). The presence of the CMX protein was detected by incubation with the substrate diaminobenzidine (Roche, Germany).

### Animals

Specific-pathogen-free female BALB/c mice (4–8 weeks old), purchased from CEMIB-Unicamp, were maintained in ABSL-2 racks adapted with an HEPA air filter, with water and food provided *ad libitum*, at the animal-care facility of the Institute of Tropical Pathology and Public Health at University Federal of Goiás. The temperature was maintained at 20–24°C with a relative humidity of 40%–70%, and 12 h light/dark cycles. This study was carried out in strict accordance with the recommendations in the Guide for the Care and Use of Laboratory Animals of the Committee of Sociedade Brasileira de Animais de Laboratório (SBAL) and approved by the Research Ethical Committee of Federal University of Goiás (Permit Number: 229/11).

### Immunization

Frozen stocks of recombinant mc^2^-CMX or mc^2^ containing empty pLA71 were thawed and the mice were inoculated subcutaneously with 10^7^ colony-forming units (CFU). The control mice were inoculated with sterile saline. The mice were immunized twice with 100 µL volumes, with an interval of 15 days between immunizations. For protection experiments a group of BALB/c mice were vaccinated subcutaneously with 10^6^ CFU of BCG-Moreau, forty-five days previous to the *Mtb* infection. All experiments were performed thrice.

### *Mycobacterium Tuberculosis* Challenge and Bacterial Load Determination

*Mycobacterium tuberculosis* strain H37Rv was grown to mid-log phase in 7H9 medium containing OADC and 0.05% Tween 80 and then frozen in aliquots in 20% glycerol at –80°C until required. Mice were infected intravenously with 100 µL of 10^7^ CFU/mL. The bacterial loads were determined by plating whole or partial organ homogenates onto nutrient 7H11 agar supplemented with OADC. The colonies were counted after incubation for 3–4 weeks at 37°C.

### Serum Collection

Serum samples were collected from the immunized mice 30 and 45 days after the second immunization. The samples were incubated for 1 h at 37°C, centrifuged at 1,200g at 4°C for 15 min to separate the serum, and stored at –20°C.

### Enzyme-linked Immunosorbent Assay (ELISA)

The ELISA was performed as optimized by Sousa et al. (2012) [15]. Briefly, 96-well polystyrene plates (Nunc®) were coated with 10 µg/mL CMX recombinant fusion protein diluted in 0.05 M sodium carbonate/bicarbonate buffer, and incubated at 4°C for 16 h. The wells were blocked with PBS containing 1% skim milk. The serum samples were diluted 1∶800, added to the wells, and incubated for 2 h at 37°C. Biotin-conjugated antibodies (anti-IgG1 or anti-mouse IgG2a; Pharmingen®) diluted 1∶5,000 were added to the plates, which were then incubated for 1 h at 37°C. Streptavidin peroxidase, diluted 1∶1,000, was added and the plates were incubated again for 1 h at 37°C. After incubation with the substrate solution, the absorbance at 492 nm was read on an ELISA reader (Labsystems Multiskan Thermo®).

### CMX-specific Responses in Lung and Spleen Cells

Thirty days after the final immunization and 30 days after the *Mtb* challenge, the mice were killed and their lungs and spleens removed aseptically. The cells were separated with a Potter tissue homogenizer (Corning, USA). The erythrocytes were lysed with Gey’s solution. The cell concentration was adjusted to 1×10^6^ cells/mL and plated in a 24-well plate. The splenocyte and lung homogenates were stimulated with ConA (10 µg/mL) or CMX (10 µg/mL) or not stimulated. After incubation in a 5% CO_2_ incubator for 2 h at 37°C_,_ monensin solution (eBioscience) was added to the cells, which were then incubated for a further 4 h. The cells were then stained for CD4^+^, CD8^+^, IFN-γ, IL2, IL17, and TNF-α using the BD Cytofix/Cytoperm Kit according to the manufacturer’s instructions. The cells were acquired with a BD Biosciences FACSCanto II flow cytometer (Albert Einstein College of Medicine and Laboratório de Nanotecnologia Farmacêutica e Sistema de Liberação de Fármacos, UFG), and the data were analyzed using the FlowJo 8.7 software. Thirty thousand lymphocytes were acquired from each sample.

### Statistical Analysis

The results were tabulated with Excel (version 14.3.4, 2011 for Mac) and the Prism software (version 5.0a, GraphPad). The differences between groups were assessed with a two-tailed Student’s *t* test after a nonparametric (Mann–Whitney U) test. The results were considered significantly different when p<0.05.

## Results

### mc^2^-CMX Vaccine is Stable *in vivo*

**Figure 1:**
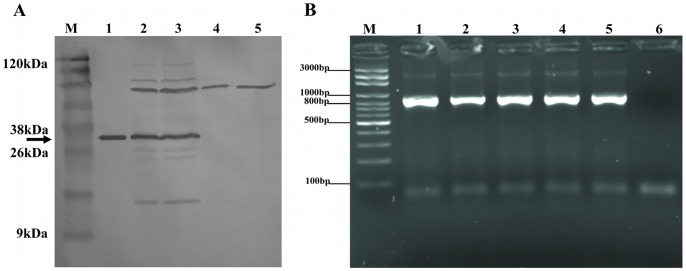
Recombinant mc^2^-CMX vaccine containing pLA71/CMX DNA expressed the recombinant protein and persisted in mice. (**A**) The recombinant CMX protein (38 kDa) expressed from the mc^2^-CMX vaccine was only detected when analyzed with an anti-CMX polyclonal antibody. Cross-reactivity with Ag85 and MPT-51 proteins was also observed. Lane M: BlueStep™ Broad Range Protein Marker (Amresco); lane 1: purified recombinant CMX protein; lanes 2 and 3: mc^2^-CMX; lanes 4 and 5: mc^2^. (**B**) To check that the live vaccine survived *in vivo* and retained the plasmid, spleen homogenates from vaccinated mice were plated on antibiotic-containing medium and the retrieved colonies were tested by PCR for the presence of the CMX fusion gene. All the isolated colonies showed a positive PCR. Lane M, Axygen 100 bp DNA Ladder (Biosciences); lanes 1–5: colonies of bacteria recovered and tested by PCR; lane 6, negative PCR control.

The recombinant *M. smegmatis* vaccine transformed with pLA71 containing CMX-encoding DNA expressed the CMX fusion protein (Figure 1). The expected size of the recombinant CMX protein (∼36 kDa) was determined with a polyclonal anti-CMX antibody when the protein extracted from mc^2^-CMX was analyzed. The Ag85, MPT51, and HspX bands were also detected with the polyclonal antibody directed against CMX (Figure 1A).

To check whether the live vaccine persisted *in vivo* without antibiotic selection and retained the plasmid, bacteria were recovered from the spleens of the vaccinated animals and grown in medium with or without kanamycin. The retrieved colonies were then tested with PCR for the presence of the plasmid containing the insert. We recovered recombinant vaccine for up to eight days after vaccination. All the colonies isolated were PCR-positive for the CMX fusion gene (Figure 1B).

### mc^2^-CMX Induces Both Humoral and Cellular Immune Responses

**Figure 2:**
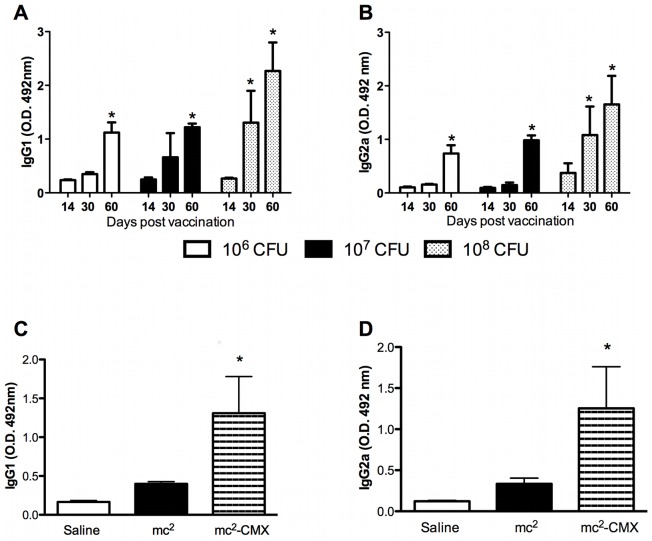
Induction of CMX-specific IgG1 and IgG2a by the mc^2^-CMX vaccine. (A and B) Animals were immunized with a single dose of different concentrations of the mc^2^-CMX vaccine and their sera were tested with an indirect ELISA at the specified time points (n = 4; *p<0.05: differences were compared on day 14 after vaccination). (C and D) Groups of 4–6 mice were vaccinated twice with 10^7^ CFU of mc^2^, mc^2^-CMX, or saline. Thirty days after vaccination, their blood was collected and their sera tested for CMX-specific IgG1 and IgG2a. *p<0.05. The results are representative of three independent experiments.

To determine the optimal dose of mc^2^-CMX for use as a vaccine, groups of mice were vaccinated with a single dose containing 10^6^, 10^7^, or 10^8^ CFU of the mc^2^-CMX vaccine, and the specific humoral immune response was assessed. As shown in Figure 2 (A and B), all vaccine doses induced specific IgG1 or IgG2a responses to CMX. Therefore, we selected the intermediate dose (10^7^ CFU) for the prime-boost strategy. To check the immunogenicity of this strategy, the sera and peripheral blood mononuclear cells from immunized mice were analyzed. IgG1 and IgG2a levels were measured and showed that a specific humoral immune response to CMX was induced for both IgG isotypes: IgG1 and IgG2a (Figure 2C and D, respectively; p<0.05).

**Figure 3:**
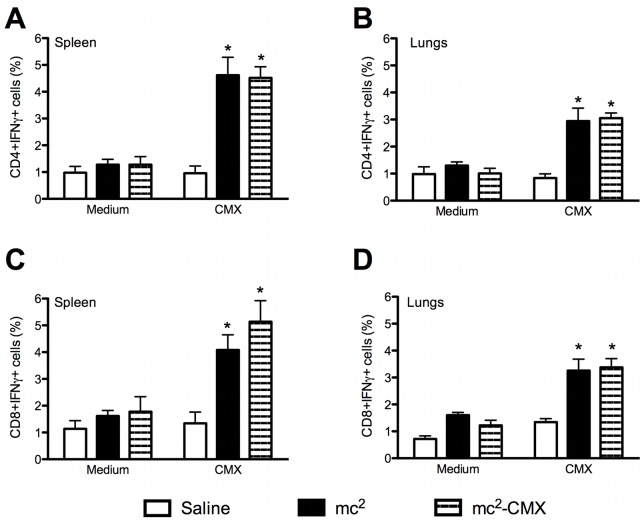
Induction of specific cellular responses to *ex vivo* stimulation with CMX. Lung and spleen single-cell suspensions from vaccinated and control mice were restimulated with CMX or medium, and the CD4^+^ and CD8^+^ IFN-γ-positive T cells were analyzed by flow cytometry. Lymphocytes were selected by their size and granularity and the CD4^+^ cells were gated and analyzed for IFN expression. A and B show CD4^+^ IFN-γ-positive cells from the spleen and lungs, respectively. C and D show CD8^+^ IFN-γ-positive cells (n = 4; *p<0.05) from the spleen and lungs, respectively. The results are representative of two independent experiments.

Because the Th1 immune response is associated with protection, we investigated whether the subcutaneous live vaccine could induce a specific Th1 response in the spleens and lungs of vaccinated mice. Gated CD4^+^ T cells, selected from cells with a live lymphocyte phenotype, were evaluated for the expression of IFN-γ after *ex vivo* restimulation with CMX (Figure 3A and B). Both the mc^2^ and mc^2^-CMX vaccines induced IFN-γ-positive CD4^+^ T cells in the lungs and spleens of the vaccinated animals (p<0.05).

It has been shown that CD8^+^ T cells also contribute to the protective cytokine profile. As shown in Figure 3C and D, CD8^+^ splenocytes showed specific responses to CMX when mc^2^ and mc^2^-CMX were used as vaccines.

**Figure 4:**
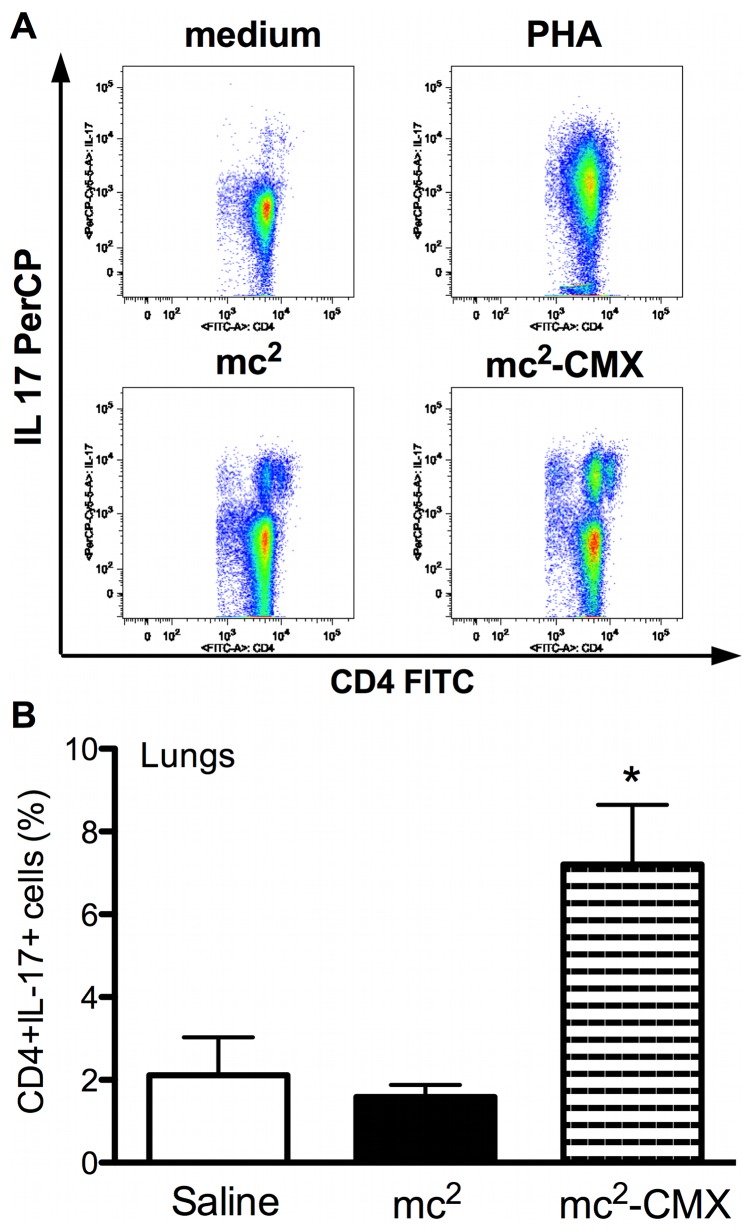
Th17 induction after vaccination with mc^2^-CMX. (A) Representative dot plots of spleen CD4^+^ T cells expressing IL17. Spleens from mice vaccinated with saline, mc^2^, or mc^2^-CMX were evaluated after *ex vivo* stimulation with medium, phytohemagglutinin (PHA), or recombinant CMX. No differences were found between the mc^2^ and mc^2^-CMX groups. (B) Lung cells from saline-, mc^2^-, and mc^2^-CMX-treated animals were restimulated *ex vivo* with recombinant CMX, and the CD4^+^ T cells were analyzed for IL-17 expression (n = 4; *p<0.05, different from the saline-vaccinated group). The results are representative of two independent experiments.

Th17 cells have been associated with a persistent memory phenotype in a murine model of TB vaccination [20]. We tested this cell population in both spleen and lung homogenates. Although we saw no difference in the induction of CMX-specific CD4^+^IL17^+^ responses in the spleen (Figure 4A), there was a distinct and specific response to CMX cells in the lungs of the mc^2^-CMX-vaccinated animals (Figure 4B).

**Figure 5:**
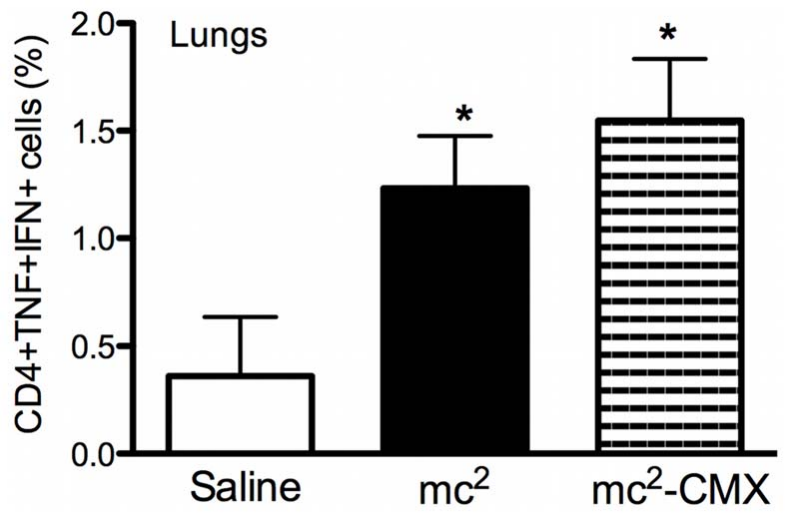
Induction of high levels of CD4^+^ T cells doubly positive for TNF-α and IFN-γ. Lung cells from saline-, mc^2^-, and mc^2^-CMX-treated animals were restimulated *ex vivo* with recombinant CMX, and the CD4^+^ T cells were analyzed for TNF-α and IFN-γ expression (n = 4; *p<0.05, different from the saline-vaccinated group). The results are representative of two independent experiments.

Polyfunctional T lymphocytes expressing protective cytokines are also associated with protection against TB. We assessed the induction of lung CD4^+^ T cells positive for both TNF-α and IFN-γ after this vaccination schema. Again, the animals vaccinated with mc^2^ or mc^2^-CMX showed the induction of specific double-positive CD4^+^TNF-α^+^/IFN-γ^+^ lymphocytes (Figure 5), although the greatest induction occurred in the mc^2^-CMX-immunized group (Figures S1 and 5).

**Figure 6:**
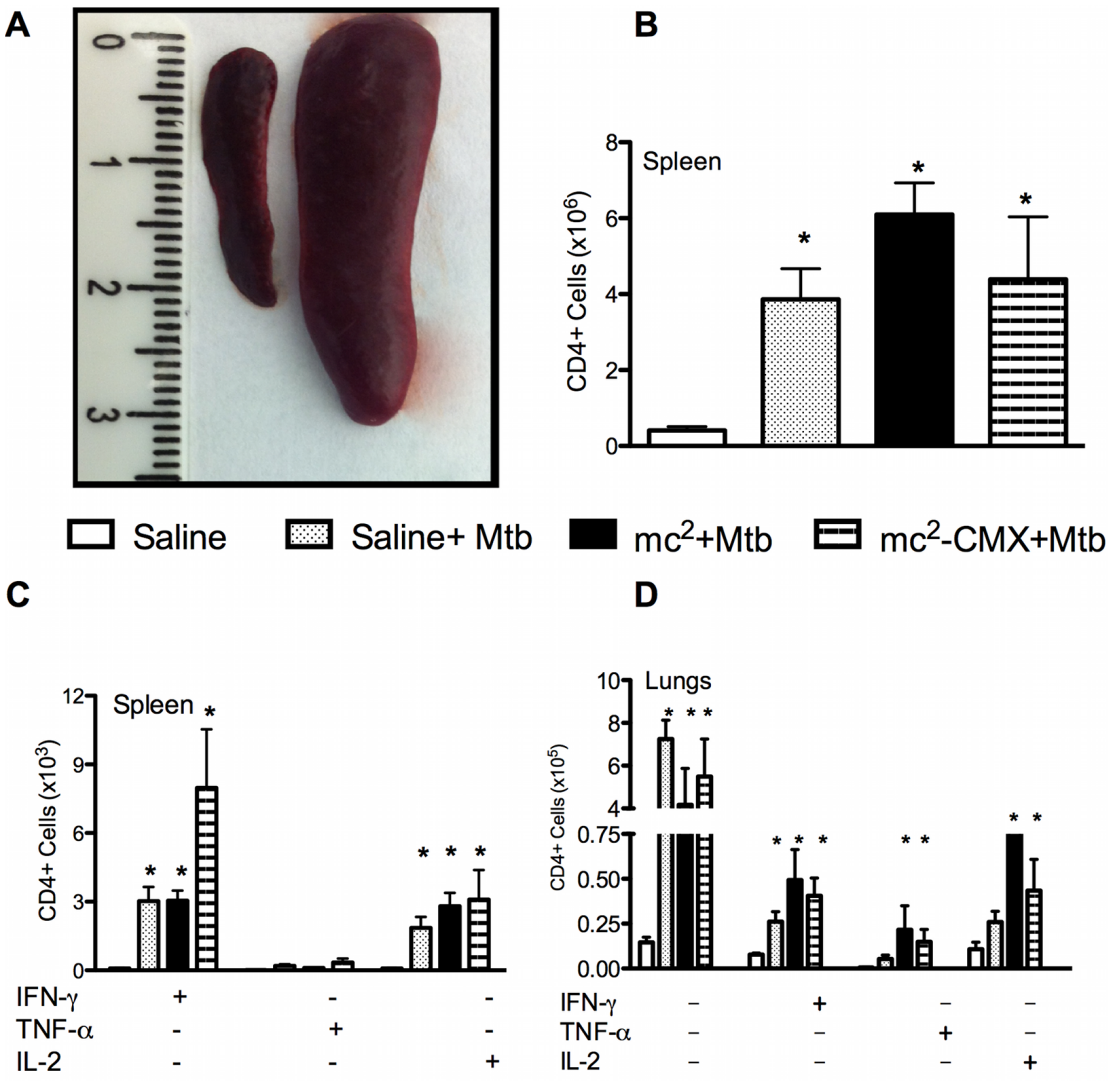
Challenge with *Mtb* induced massive splenomegaly and increases in the CD4^+^ T-cell populations. (A) Representative photographs of the spleens from control and *Mtb*-infected animals. Mice were challenged with *Mtb* and 45 days after infection, their spleens were collected for flow-cytometric analysis. (B) Total CD4^+^ T cells from mice vaccinated with saline, mc^2^, or mc^2^-CMX and infected with *Mtb*. (C) Spleen CD4^+^ T-cell response to *ex vivo* stimulation with CMX. CD4^+^ T cells selected with lymphocyte gating for IFN-γ, TNF-α, and IL2 were quantified. (D) Lung CD4^+^ T-cell response to *ex vivo* stimulation with CMX (n = 4; *p<0.05, different from the saline-vaccinated group). The results are representative of two independent experiments.

We then investigated whether challenging the animals with *Mtb* affected the immune response elicited by vaccination. After intravenous infection with *Mtb*, splenomegaly was observed in all groups, with an almost fivefold increase in the total number of CD4^+^ T cells (Figures 6A and B). Whereas the total numbers of these cells increased in all the groups tested, the greatest increase in CD4^+^IFN-γ^+^ cells was observed predominantly in animals vaccinated with mc^2^-CMX (Figure 6C). The total numbers of CD4^+^ T cells and CD4^+^IFN-γ^+^ T cells also increased in the lungs of the challenged animals (Figure 6D), but similar increases in the double-positive CD4^+^TNF-α^+^ and CD4^+^IL2^+^ cells were only observed in the vaccinated and challenged groups.

**Figure 7:**
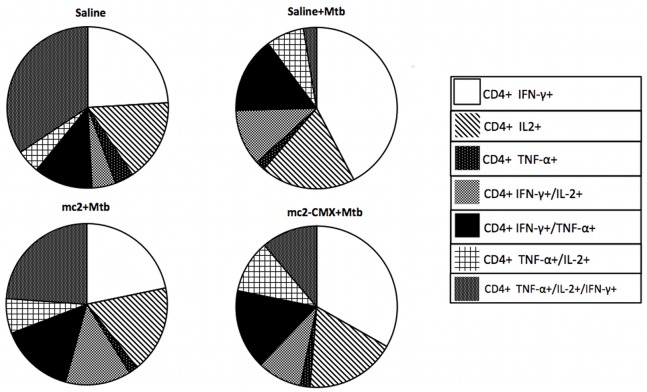
Reduction in splenic polyfunctional CD4^+^ T cells after *M. tuberculosis* infection prevented by mc^2^-CMX vaccination. Mice were challenged with *Mtb* and 45 days after the infection, their spleens were collected for flow-cytometric analysis. CD4^+^ T cells selected with lymphocyte gating for IFN-γ, TNF-α, and IL-2 were quantified. Populations were defined as singly, doubly, or triply positive for the cytokines.

Triple-positive polyfunctional CD4^+^ T cells were evaluated in the spleen, and although *Mtb* induced increases in spleen CD4^+^IFN-γ^+^ and CD4^+^IL2^+^ cells, the infection significantly reduced the frequencies of triple-positive CD4^+^TNF-α^+^IFN-γ^+^ cells. Previous vaccination with mc^2^-CMX prevented this dramatic reduction in triple-positive cells, and doubled the frequency of CD4^+^TNF-α^+^IL2^+^ cells (Figure 7).

**Figure 8:**
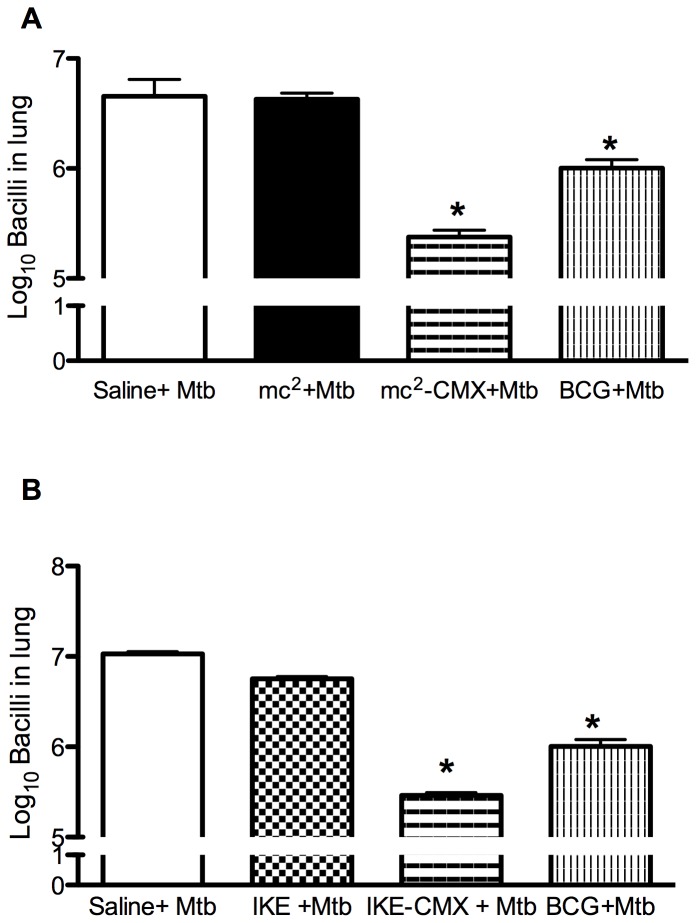
Vaccination with mc^2^-CMX or IKE-CMX reduced the bacterial load in *Mtb*-challenged mice. Groups of mice vaccinated with saline, mc^2^, mc^2^-CMX, or BCG (A), and then vaccinated with saline, IKE, IKE-CMX, or BCG (B) were challenged with *Mtb* 45 days after immunization. Thirty days after infection, their lungs were collected and CFU counted. *p<0.05 when compared with the saline+*Mtb* group.

We then assessed whether vaccination with mc^2^-CMX protected the mice against *Mtb* challenge. The mc^2^-CMX vaccine reduced the number of bacteria recovered from the lungs by approximately 1 log unit, compared with both the nonvaccinated (saline control) and mc^2^-vaccinated groups. Surprisingly mc^2^-CMX vaccinated group showed 0.5 Log of CFU reduction when compared to BCG vaccinated group (Figure 8A).

We also investigated whether the recombinant fusion protein could induce protection against *Mtb* in the context of the safer *M. smegmatis* background in the same way as was shown here. IKE *M. smegmatis* was transformed with pLA71 containing CMX (IKE-CMX) and after mice were subjected to the same vaccination scheme, they were challenged with *Mtb.* IKE alone reduced the bacterial load compared with that in the infection-only control group, as shown previously [19], whereas the IKE-CMX vaccine reduced the bacterial load even further when compared to BCG vaccination (Figure 8B; p<0.05).

**Figure 9:**
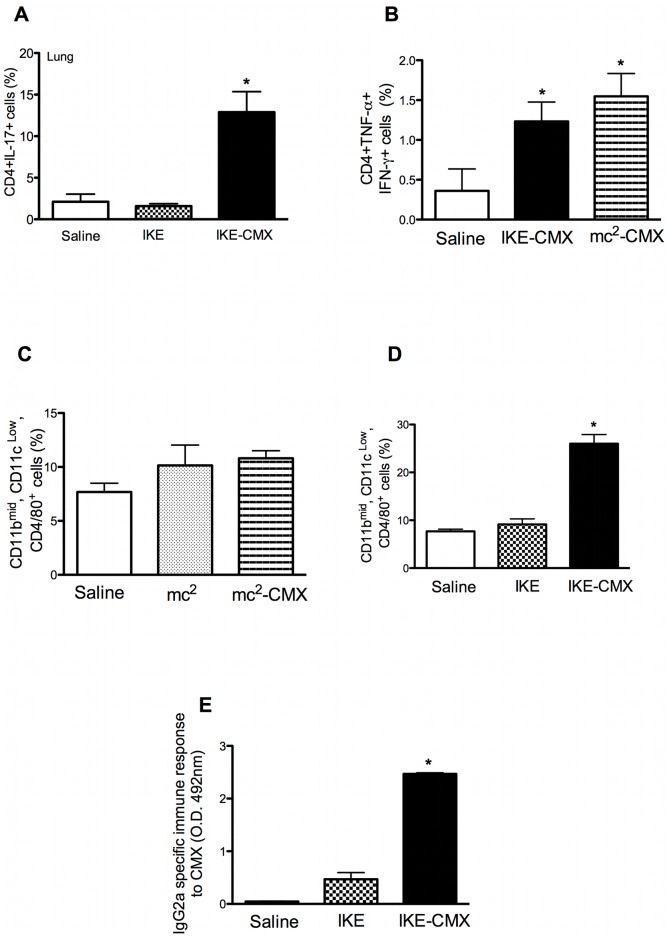
IKE-CMX vaccination activates CD4^+^ T cells, macrophages, and the IgG2a immune response. Groups of mice vaccinated with saline, IKE, or IKE-CMX (A) and then vaccinated with saline, IKE-CMX, or mc^2^-CMX (B) were challenged with *Mtb* 45 days after immunization. Thirty days after infection, their lungs were collected. CD4^+^ T cells selected with lymphocyte gating for IL-17 (A), or IFN-γ and TNF-α (B) were quantified. Lung macrophages, defined as CD11b^mid^, CD11c^low^, and F4/80^+^, were quantified after mc^2^-CMX (C) or IKE-CMX (D). ELISA was used to detect CMX-specific serum IgG2a after the IKE-CMX vaccination scheme (E).

Once we had established that both vaccines reduced the bacterial load in the lung and spleen, and that the protection induced by IKE-CMX was similar to that induced by mc^2^-CMX, we analyzed the specific immune response to CMX and macrophage activation. As shown in Figure 9, IKE-CMX induced the same frequencies of both CD4^+^IL17^+^ and CD4^+^TNF-α^+^IFN-γ^+^ cells as mc^2^-CMX (A and B) and similar results were observed for all polyfunctional and single-positive CD4^+^ T cells (data not shown). However, although there was no difference in the proportion of spleen macrophages (CD11b^mid^, CD11c^low^, CD4^+^/80^+^ cells) after mc^2^-CMX vaccination (Figure 9C), IKE-CMX induced a robust increase in the frequency of this cell population (Figure 9D). Similar to the results obtained with mc^2^-CMX, the IKE-CMX vaccine induced a specific humoral immune response to CMX (Figure 9E).

## Discussion

In this study, a vaccine composed of a nonpathogenic mycobacterium expressing a recombinant fusion protein composed of epitopes from *Mtb* antigens (CMX) induced a significant immune response in mice, affording them protection against *Mtb* challenge.

*Mycobacterium smegmatis* is a nonpathogenic member of the genus *Mycobacterium* that grows rapidly and is easily manipulated to produce recombinant bacteria. Consequently, it is a valuable host for use as a live vaccine against TB [19], [21]. *Mycobacterium smegmatis* does not survive long within the cellular host environment because it is readily eliminated by phagosomal proteases [22]. Therefore, unlike pathogenic mycobacteria, these bacteria are processed upon infection and presented very rapidly by antigen-presenting cells [23]. Our results show that *M. smegmatis* expressing CMX survived for up to 8 days after vaccination, indicating that this period of time was sufficient to induce a specific immune response to CMX. This result corroborates the notion that nonpathogenic mycobacteria induce macrophages that produce higher levels of innate related cytokines than those induced by pathogenic mycobacteria [24]. They also activate dendritic cells by upregulating class I major histocompatibility complex and costimulatory molecules [25].

Another interesting characteristic of this bacterium, making it a suitable vaccine vector, is its nontoxicity in immunodeficient animal models lacking NK or T cells, as seen in the studies by Young et al. This suggests that this vehicle is safe for use as a vaccine in immunocompromised individuals [26], [27]. Consequently, *M. smegmatis* has been used to express recombinant proteins in several studies. A particular *M. smegmatis* recombinant vaccine expressing a fusion protein containing ESAT-6 and CFP10 induced higher humoral and cellular immunity than *M. bovis* BCG vaccination in a mouse model [21]. Similarly, Yi et al. demonstrated that a recombinant *M. smegmatis* vaccine that produced a fusion of murine IL12 and human granulysin in BALB/c mice induced an efficient protective Th1 response, including high levels of IFN-γ and IL12, similar to that induced by *M. bovis* BCG [28]. Here we showed that recombinant *M. smegmatis* vaccines expressing CMX in two different backgrounds (mc^2^ and IKE) were able to reduce the bacterial load of lungs from *Mtb* infected animals, when compared to *M. bovis* BCG vaccinated BALB/c mice.

We have shown that our recombinant vaccine mc^2^-CMX is capable of inducing a specific humoral response to CMX in three different vaccination schemes. This response probably reflects the immunogenicity of the recombinant fusion protein when used in this vaccine vector. The results of our group and others have confirmed that Ag85C, MPT51, and HspX are *Mtb* antigens with immunogenic and antigenic properties represented by the epitopes expressed in the recombinant fusion protein [6], [12], [14], [15], [29]–[31]. Because this protein specifically induced both IgG2a and IgG1, we can infer that both Th1 (IFN-γ) and Th2 (IL4) subpopulations of cells were induced [32], [33].

The CMX-specific response of CD4^+^ and CD8^+^ T cells *ex vivo* showed that both vaccines, with or without CMX expression, induced IFN-γ-positive CD4^+^ and CD8^+^ T cells. The ability of mc^2^ alone to induce a response to CMX might seem unexpected, but it can be explained by the fact that *M. smegmatis* contains genes that encode Ag85C, MPB-51, and a hypothetical protein MSMEG_3932, with high similarities (up to 86%) to the partial sequences of *Mtb* Ag85C, MPT51, and HspX, respectively, which were used to construct the recombinant fusion protein [15]. Further evidence that mc^2^ expresses those individual proteins and that they cross-react with polyclonal antibodies directed against CMX is presented in Fig. 1A. Despite these CD4 and CD8 T cells responses in both vaccinated groups (mc^2^ and mc^2^-CMX), only mice vaccinated with mc^2^-CMX showed better protection. This raise the question addressed currently if interferon gamma alone is responsible for protection and could be used as surrogate marker for protection once individuals with active TB presents specific interferon producers T cells [34], [37].

Here, we have shown that vaccination with mc^2^ induced high levels of CD4^+^ T cells in spleens expressing IL17 that were not specifically generated by the presence of CMX. Nevertheless, lung Th17 CD4^+^ T cells were generated and responded specifically to CMX. Although it has recently been shown that IL17 and IL22 do not contribute to *Mtb* control in mice [34], several studies have suggested that *Mtb* vaccines generate long-lasting memory Th17 cells [20], [35]. However, to our knowledge, no previous work has investigated whether the expansion of lung specific Th17 cells before challenge with *Mtb* contributes to a protective effect. Additionally in this work, we showed that higher levels of Th17 cells in the spleens of vaccinated mice were not associated with protection because mice vaccinated with mc^2^ did not reduced the bacterial load. Prompting us to hypothesized that maybe only specific lung Th17 cells were important for protection [20], [35].

Vaccination with mc^2^-CMX induced a huge increase in CD4^+^IFN-γ^+^ cells specific to CMX in both the spleen and lungs. We speculate that the mobilization of specific Th1 and Th17 cells to the lungs before challenge with *Mtb* may contribute to the protective effect by increasing the activities of phagocytes.

IFN-γ and TNF-α act synergistically in mediating the killing of intracellular pathogens, increasing the bactericidal activity of macrophages, and preventing the spread of bacilli [36]–[38]. IL2 is necessary for the secondary expansion of memory T cells and therefore in the generation of persistent vaccine-induced immunity [39], [40]. The differences between the types of cytokines produced by individual cells have profound implications for their ability to mediate memory or effector functions. Therefore, the ability to induce persistent *Mtb*-specific T-cell responses is an important factor in the development new vaccines [41]. Double-positive CD4^+^ T cells have been shown to express CCR7 in their membranes and can proliferate in the presence of the antigen, and are therefore characterized as central memory cells [42]–[44]. Although the results presented here do not show this cell-surface marker, we speculate that the CD4^+^TNF-α^+^IL-2^+^ cells were memory cells.

Polyfunctional specific CD4^+^ T cells were detected in animals vaccinated with mc^2^ and this response increased after immunization with mc^2^-CMX, which induced the highest numbers of CD4^+^TNF-α^+^IFN-γ^+^ cells. Several studies have shown that the induction of polyfunctional T cells correlates directly with protection from intracellular pathogens, such as viral infections, parasites, and chronic bacterial infections [44]–[48]. It also appears that the highest frequency of polyfunctional specific CD4^+^ T cells co-expressing IL2, IFN-γ, and TNF-α is associated with the best outcomes of different vaccine formulations directed against *Mtb* [47], [48]. It is very important to note that in this study, *Mtb* infection reduced the numbers of polyfunctional CD4^+^ T cells and that this phenomenon was prevented by the mc^2^-CMX vaccine. Although our results are consistent with those of previous studies, some controversial findings regarding polyfunctional CD4^+^ T cells clearly indicate the need of further research to understand their role in TB [47], [49]–[51].

We could not determine whether the immune responses to Ag85C, MPT51, and HspX were directly responsible for the reduced bacterial load observed in our results. Those three antigens are very important for *Mtb* survival, both *in vivo* and *in vitro*. Ag85C is a member of the family of proteins responsible for mycolic acid synthesis, a critical cell wall component [52], MPT51 is an α-hydrolase and has been shown to bind to host fibronectin [53], and HspX is a secreted protein produced by bacteria under stress conditions [54]. Therefore, the specific immune response induced to those antigens and others expressed by mc^2^ and other vaccines may have impaired bacterial growth in the organs examined. It is important to notice that both recombinant vaccines expressing CMX presented better protective performance than BCG, favoring our conclusions.

The expression of CMX in an IKE background increased the efficacy of our recombinant vaccine. The development a vaccine in a genetic background that has already been shown to be safe for immunocompromised mice or patients is essential (19). IKE-CMX induced almost double the number of CD4^+^IL17^+^ cells, which are associated with the memory response (20). There was also a huge increase in spleen macrophages (CD11b^mid^, CD11c^low^, CD4^+^/80^+^), suggesting that this recombinant strain of *M. smegmatis* is handled by the innate immune response, resulting in the generation of improved effector and protective memory immune responses.

In conclusion, the expression of CMX improved the immune responses and the protection induced when *M. smegmatis* mc^2^ or IKE were used as a vaccine against TB.

## Supporting Information

Figure S1 **Flow cytometry quadrant sets to quantify CD4^+^IFN-γ^+^TNF-α^+^ T spleen cells.** Representative dot plots of spleen CD4^+^ T cells expressing IFN-γ and TNF-α are shown. Lymphocytes were gated based upon size and granulocity. Then CD4+ were further gated and analyzed for cytokines expression. Quadrant sets were set using panels with anti- CD4 FITC and rat IgG1-APC and rat IgG1- PE and then compared to the panels using anti- CD4 -FITC, anti-IFN-**γ -**APC and anti-TNF-α- PE.
(TIFF)
