# Supplementary figures and images for: Prime–Boost with Mycobacterium smegmatis Recombinant Vaccine Improves Protection in Mice Infected with Mycobacterium tuberculosis
Source: PLoS One. 2013 Nov 8;8(11):e78639. doi: 10.1371/journal.pone.0078639 (PMC3826754; doi:10.1371/journal.pone.0078639)

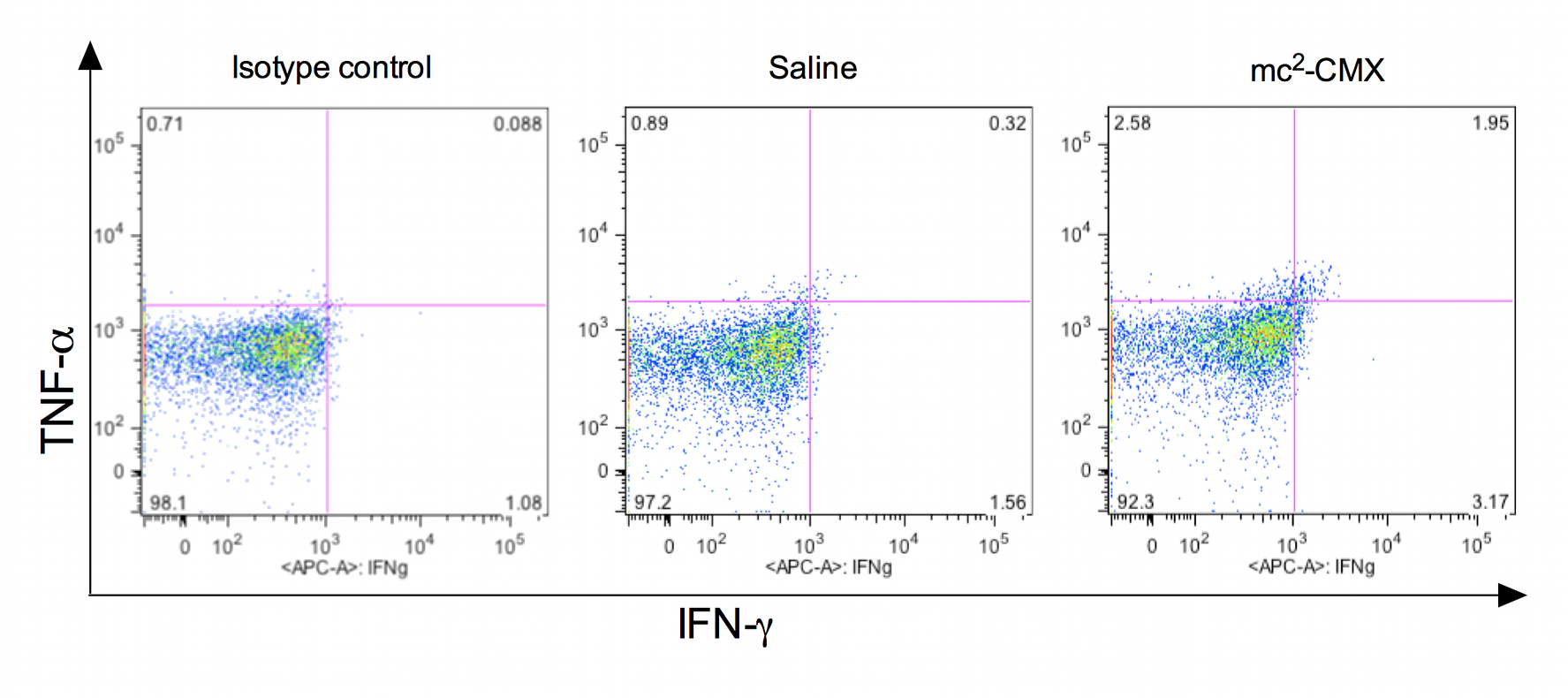

Supplement: Figure S1 — Flow cytometry quadrant sets to quantify CD4+IFN-γ+TNF-α+ T spleen cells. Representative dot plots of spleen CD4+ T cells expressing IFN-γ and TNF-α are shown. Lymphocytes were gated based upon size and granulocity. Then CD4+ were further gated and analyzed for cytokines expression. Quadrant sets were set using panels with anti- CD4 FITC and rat IgG1-APC and rat IgG1- PE and then compared to the panels using anti- CD4 -FITC, anti-IFN-γ -APC and anti-TNF-α- PE. (TIFF) [file pone.0078639.s001.tif]
